# Antioxidant effect of mono- and dihydroxyphenols in sunflower oil with different levels of naturally present tocopherols

**DOI:** 10.1002/ejlt.201200293

**Published:** 2013-05-17

**Authors:** Iveta Hrádková, Roman Merkl, Jan Šmidrkal, Jan Kyselka, Vladimír Filip

**Affiliations:** Department of Dairy, Fat and Cosmetic Science, Institute of Chemical TechnologyPrague, Czech Republic

**Keywords:** Alkyl esters of phenolic acids, Antioxidant activity, Lipophilic antioxidants, Phenolic acids, Tocopherols

## Abstract

Antioxidant properties of mono- and dihydroxyphenolic acids and their alkyl esters were examined, with emphasis on the relationship between their molecular structure and antioxidant activity. Test media with different tocopherol level were used for determining the oxidative stability: original refined sunflower oil (total tocopherols 149.0 mg/kg), partially tocopherol-stripped sunflower oil (total tocopherols 8.7 mg/kg) and distilled fatty acid methyl esters (FAME) as a tocopherol-free medium. The chemical reaction of tocopherols with diazomethane tested for the purpose to eliminate their antioxidant activity failed due to the negligible degree of methylation of hydroxyl group in the tocopherol molecule. Caffeic acid and protocatechuic acid (3,4-dihydroxyphenolic acids) and their alkyl esters were found to be more active antioxidants than monohydroxyphenolic acid (*p*-hydroxybenzoic acid), 2,5-dihydroxyphenolic acid (gentisic acid), 3-methoxy-4-hydroxyphenolic acids (vanillic and ferulic acids) and their corresponding alkyl esters. Naturally present tocopherols in refined sunflower oil proved to have a synergistic effect on gentisic acid but not on its alkyl esters. In contrast, tocopherols showed an antagonistic effect on alkyl esters of caffeic acid, because their protection factors decreased with increasing level of tocopherols in the test medium. Moreover, the antioxidant activity of these alkyl esters decreased with increasing length of their alkyl chain in conformity with the polar paradox hypothesis.

**Practical applications:**

Tocopherols as naturally present antioxidants influence considerably the antioxidant activity of other antioxidants added to plant oils used as a test medium. Distilled fatty acid methyl esters prepared from refined sunflower oil may serve as an optimal tocopherol-free test medium. Some alkyl esters of phenolic acids were evaluated to be applicable as natural more lipophilic antioxidants in comparison with phenolic acids.

## 1. Introduction

Lipid oxidation is responsible for a consequent decrease in nutritional and sensory quality of lipid-containing products. Their stability is directly associated with addition of suitable antioxidants. In the last years, there is a growing interest in natural antioxidants found in plants because of the world-wide trend toward the use of natural additives in food and cosmetics [1]. Phenolic acids are frequently studied because of their anti-inflammatory, anti-allergic, antimicrobial, anticarcinogenic, antiviral, and antioxidant effects. Phenolic acids occur in oilseeds (sinapic acid in rapeseed, *p*-hydroxybenzoic and caffeic acid in soya beans, chlorogenic acid in sunflower seed) [2], herbs (ferulic acid in lavender, caffeic acid in oregano, caffeic acid in lemon balm, rosmarinic acid in rosemary) [3], fruit (gallic and caffeic acid in blackberries, *o*-, *m*-, and *p*-hydroxybenzoic acid in cranberries), and vegetable (hydroxycinnamic acid derivatives and *p*-hydroxybenzoic acid in carrot, chlorogenic acid in potato) in unbound or bound forms (as glycosides or esters) [4].

Polarity of phenolic antioxidants influences their effect. Potter et al. [5] reported that more polar antioxidants are effective in nonpolar system of bulk oil and more lipophilic antioxidants are effective in polar system of oil-in-water emulsion. Polar antioxidant (such as Trolox and ascorbic acid) accumulates on oil–air interfaces in nonpolar system and thus protects oil against oxidation. In contrast, nonpolar antioxidants (such as α-tocopherol and ascorbyl palmitate) are dissolved in the oil phase. This paradoxical behavior of antioxidants is known as the polar paradox [6].

Phenolic acids act as primary antioxidants, exhibiting antioxidative potential by donating a hydrogen atom for breaking the free radical chain [7]. The molecular structure (position and number of hydroxyl groups) of phenolic acids has a considerable effect on their antioxidative properties. Phenolic acids with two (protocatechuic acid – 3,4-dihydroxybenzoic acid; caffeic acid – 3,4-dihydroxycinnamic acid) and more hydroxyl groups in a molecule (gallic acid – 3,4,5-trihydroxybenzoic acid) are more effective antioxidants than phenolic acids with only one hydroxyl group (3- or 4-hydroxybenzoic acid). The position of hydroxyl groups is also important, for example, 2,5-dihydroxybenzoic acid has lower antioxidant activity than 2,3-dihydroxybenzoic acid. Methylene group (3,4-dihydroxyphenylacetic acid) or ethylene group (caffeic acid) inserted between a phenyl ring and carboxylic group brings about the significant changes in antioxidant activity [8]. Derivatives of benzoic acid (*p*-hydroxybenzoic, vanillic, syringic, protocatechuic acid) have weaker antioxidant properties than the corresponding analogs of cinnamic acid (*p*-coumaric, ferulic, sinapic, caffeic acid) [9].

Alkyl esters of phenolic acids are known not only as efficient antioxidants but also as antimicrobial compounds [10]. Alkyl esters of caffeic and dihydrocaffeic acid have higher scavenging effects on DPPH (2,2-diphenyl-1-picrylhydrazyl) radicals than caffeic acid, while dihydrocaffeic acid shows maximal scavenging activity. This can be caused by different conformation of molecules. Dihydrocaffeic acid is connected with an aromatic ring by single bond and phenyl group can rotate flexibly, whereas rotation of phenyl group with esterified carboxylic group can be restrained. Caffeic acid has coplanar conformation [11]. Alkyl esters of rosmarinic and chlorogenic acid show so-called cut-off effect in oil-in-water emulsions. Their antioxidant activity increases with elongation of alkyl chain to critical point in homologous series of alkyl esters. Then, antioxidant activity rapidly decreases with further increase of the alkyl chain length due to a decrease of antioxidant concentration in water phase. Critical point of homologous series of alkyl esters of rosmarinic acid is octyl rosmarinate and chlorogenic acid is dodecyl chlorogenate [12, 13].

Antioxidant effect can be determined by using many accelerated methods. One group of such methods is based on determination of oxidation stability of oil with an antioxidant at higher temperatures (Schaal oven-storage test [14], active oxygen method, Rancimat test [15], Oxidograph test [16]). These tests predict shelf-life of samples and their results provide information about how much the added antioxidant increases their oxidative stability at room temperature.

If plant oil is used as the test medium, there is a problem with the presence of naturally occurring antioxidants (especially tocopherols), which may have interactive effects on the antioxidant activity of the antioxidants studied. Tocopherols can be removed from plant oil by column chromatography (adsorption on silica gel) [17, 18] or by methanolysis of triacylglycerols and subsequent distillation of fatty acid methyl esters (FAME) prepared from plant oil under low pressure [19].

The objective of this study was to prepare an optimal test medium for determination of antioxidant effect of mono- and dihydroxyphenolic acids and their more lipophilic esters and to examine how tocopherols naturally present in sunflower oil may affect the antioxidant activity of the used antioxidants.

## 2. Materials and methods

### 2.1 Test media for determining the oxidative stability

Three different lipid matrices were used as a test medium for determining the antioxidant activity of phenolic acids and their alkyl esters (the test media were analyzed by peroxide value [20], *p*-anisidine value [21], fatty acid, and tocopherol composition; Table 1):

**Table 1:** Peroxide value, *p*-anisidine value, fatty acid and tocopherol composition in original sunflower oil, tocopherol-stripped sunflower oil, and in FAME prepared from original sunflower oil

	OSOa)	TSOb)	FAME
Peroxide value (meq.act.O/kg)c)	2.1 ± 0.2	2.6 ± 0.1	2.2 ± 0.1
*p*-Anisidine value [1] c)	4.4 ± 0.1	4.9 ± 0.2	4.6 ± 0.1
Fatty acid (%)d)			
C16:0	6.2	6.8	6.6
C18:0	3.6	4.1	3.7
C18:1	26.4	29.1	26.6
C18:2	62.6	58.8	62.1
C18:3	1.0	0.3	0.8
Tocopherol (mg/kg)			
α	120	2.4	nd
β	10	nd	nd
γ	12	5.6	nd
δ	7	0.7	nd

nd, not detected.

a) Original sunflower oil.

b) Tocopherol-stripped oil.

c) Results are expressed as means ± SD of three samples.

d) % of total fatty acids.

#### 2.1.1 Original sunflower oil (Vegetol Gold, Oleofin a.s., Ústí nad Labem, Czech Republic)

#### 2.1.2 Tocopherol-stripped sunflower oil

Tocopherol-stripped sunflower oil was prepared according to the modified method of Waraho et al. [18]. Briefly, a glass chromatographic column (internal diameter 3.0 cm, height 35 cm) was packed with 45 g of activated Silica gel 60 (Merck KGaA, Darmstadt, Germany) dissolved in *n*-hexane. Original sunflower oil (30 g) was dissolved in 30 mL of *n*-hexane and passed through the column by eluting with 270 mL of *n*-hexane. The tocopherol-stripped sunflower oil was obtained by solvent removing with a vacuum rotary evaporator (Buchi Laboratortechnik AG, Flawil, Switzerland) at 37°C. Traces of solvent were removed by flushing with nitrogen.

#### 2.1.3 FAME prepared from original sunflower oil

FAME were prepared from original sunflower oil according to the modified method described by Rashid and Anwar [22]. Original sunflower oil was reacted with methanol in the presence of KOH (25°C, 1 h) in molar ratio 1:10:0.14. The phase containing FAME was separated from glycerol phase and washed by distilled water to neutral reaction of phenolphthalein. FAME were dried under vacuum and distilled at pressure of 3 mbar and temperature at 180°C.

### 2.2 Methylation (inactivation) of α-, γ-, and δ-tocopherol

α-, γ-, and δ-tocopherols were methylated by diazomethane (CH_2_N_2_).

Diazomethane was prepared according to Arndt [23, 24]. In the first step *N*-methyl urea reacted with NaNO_2_ in the presence of H_2_SO_4_ to form *N*-nitroso-*N*-methyl urea. In the second step *N*-nitroso-*N*-methyl urea was decomposed by KOH solution to diazomethane.

Diazomethane was dosed to 1% solution of dl-α-tocopherol (98.2%, Merck KGaA), d-γ-tocopherol (≥96.0%, Sigma–Aldrich Chemie, Steinheim, Germany) or d-δ-tocopherol (≥90.0%, Sigma–Aldrich Chemie) in diethyl ether in presence of BF_3_ (10%) as catalyst until the solution became yellow [25]. Reaction mixture was analyzed by GC-flame ionization detector (FID).

### 2.3 Antioxidants

**Figure 1:**
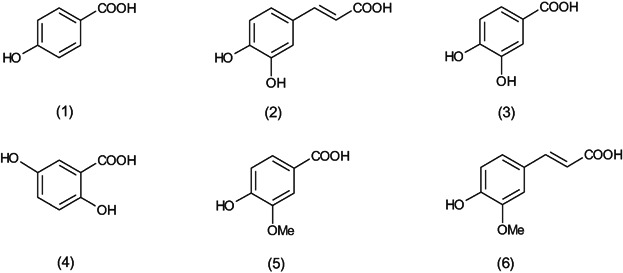
Chemical structure of *p*-hydroxybenzoic acid (1); caffeic acid (2); protocatechuic acid (3); gentisic acid (4); vanillic acid (5); ferulic acid (6).

The following phenolic acids were used (Fig. 1): *p*-hydroxybenzoic acid = 4-hydroxybenzoic acid (99%; Merck); caffeic acid = 3,4-dihydroxycinnamic acid (99%; Alfa Aesar, Karlsruhe, Germany); protocatechuic acid = 3,4-dihydroxybenzoic acid (≥97%; Sigma–Aldrich Chemie); gentisic acid = 2,5-dihydroxybenzoic acid (≥99%; Sigma–Aldrich Chemie); vanillic acid = 3-methoxy-4-hydroxybenzoic acid (98%; Merck); ferulic acid = 3-methoxy-4-hydroxycinnamic acid (98%; Merck).

### 2.4 Preparation of alkyl esters of phenolic acids

Used alcohols: methanol – 99.8%, Penta (Praha, Czech Republic); ethanol – 99.8%, Merck; propanol – 99.5%, Penta; butanol – 99.5%, Lachner s.r.o. (Neratovice, Czech Republic); hexanol – ≥ 98.0%, Sigma–Aldrich Chemie.

Alkyl esters of phenolic acids were prepared by esterification of phenolic acids by relevant alcohol, *p*-toluenesulfonic acid monohydrate was used as catalyst, according to Merkl et al. [10]. Prepared alkyl esters (≥98%) were methyl-, ethyl-, propyl-, butyl- a hexyl esters of phenolic acids (butyl ester and hexyl ester of vanillic acid were not prepared).

The content of studied antioxidants in all test media for determining their antioxidant activity was 3 mmol/kg.

### 2.5 Instrumental methods for determining of antioxidant activity

#### 2.5.1 Oxidograph method (ML Oxidograph, Mikrolab Aarhus A/S, Hojbjerg, Denmark)

A sample of oil with an added antioxidant was measured at 110°C under oxygen atmosphere and the decrease in oxygen pressure was recorded. The induction period was determined as the time interval when oxygen pressure does not decrease – IP_Oxidograph_ [16]. Induction period is mean value of three replicate analyses.

#### 2.5.2 Rancimat method (Rancimat 743, Metrohm, Herisau, Switzerland)

A sample of oil with an added antioxidant was reacted at 120°C and air was bubbled through the sample during the reaction. Volatile secondary oxidative products were carried to the demineralized water and a conductivity of water was recorded. The induction period was determined as the time interval when the conductivity does not increase – IP_Rancimat_ [26]. Induction period is mean value of three replicate analyses.

The protection factor of an antioxidant was calculated from Eq. (1):


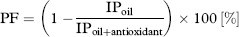
(1)

where PF is the protection factor, IP_oil_ is induction period of oil without antioxidant; IP_oil+antioxidant_ is induction period of sample of oil with antioxidant.

This equation is more suitable for determination of antioxidant effect than equation *F* = IP_oil+antioxidant_/IP_oil_ [9, 27] because antioxidant effect is in the range 0–100%, substances with prooxidant effect have negative values.

### 2.6 Fatty acid composition by GC-FID

The fatty acid profiles were analyzed using the modified methods ISO 5509:2000 and ISO 5508:1990, according to Zárubová et al. [28].

### 2.7 Tocopherol content by GC-FID

An oil sample (1.5 g) with addition of internal standard (5α-cholestane – 0.503 mg) and antioxidant (ascorbic acid – 0.3 g) was saponified by 70 mL of methanolic KOH solution (2 mol/L) for 60 min under reflux. Unsaponifiable fraction was extracted three times by diethyl ether (100 mL), washed three times by water (100 mL) and the solvent was evaporated with vacuum rotary evaporator (Buchi Laboratortechnik, Flawil, Switzerland). Two percent solution of unsaponifiable fraction in diethyl ether was prepared and then analyzed by GC-FID (Agilent Technologies 6890) with column Optima 17-TG (Macherey-Nagel, Dueren, Germany) – dimension 0.32 mm × 25 m, film thickness 0.1 μm. Helium was used as carrier gas with flow rate 1 mL/min. Sample (1 μL in split rate 1:25) was sprayed at 300°C. Analysis was isothermal (262°C). Detection was carried out at temperature 300°C (hydrogen flow was 40 mL/min, air flow was 450 mL/min, nitrogen flow as make up was 25 mL/min). Relative percentage was converted to weight percentage and expressed as mg/kg. Results are mean values of three replicate analyses.

### 2.8 Statistical analysis

All the values of induction periods and protection factors are presented as mean values ± SD, calculated from the results of three replicate analyses. Results were analyzed by applying a Student *t*-test with significance level *p* = 0.05. Microsoft Office Software (Excel, version 2003) was used to evaluate a correlation between the values of induction periods measured by the Oxidograph and Rancimat methods.

## 3. Results and discussion

Original sunflower oil was used as the test medium with higher tocopherol concentration. Tocopherol-stripped sunflower oil and FAME were used as test media with significantly lower or zero tocopherol concentration. Theoretically, methylation of pure tocopherols offered a further possibility how to remove tocopherol activity. Antioxidant activity of mono- and dihydroxyphenolic acids and their esters was determined in original sunflower oil and FAME in concentration 3 mmol/kg. The most effective antioxidants – methyl, ethyl, propyl, and butyl ester of caffeic acid were tested in original sunflower oil, tocopherol-stripped sunflower oil, and FAME for the determination of influence of different tocopherol concentration on final oxidative stability of samples.

### 3.1 Test media for determining the oxidative stability

Refined (original) sunflower oil served as the test medium with high concentration of linoleic acid. Naturally present tocopherols in the oil may affect the antioxidant activity of phenolic compounds tested (Table 1). Tocopherol concentration in original sunflower oil was lower in comparison with the data of Tasan and Demirci 29. Tocopherol loss can be explained by careless conditions during deodorization (such as high temperature, heating time, pressure, and stripping steam dosage) [30].

Tocopherols can be removed from sunflower oil by adsorption on silica gel using the technique of column chromatography. Another way to remove tocopherols from the oil is methanolysis of sunflower oil and subsequent vacuum distillation of FAME prepared from sunflower oil (the tocopherols remain in the distillation residue). FAME prepared in such a manner have a similar fatty acid composition as original sunflower oil, but tocopherols are completely removed.

#### 3.1.1 Tocopherol-stripped sunflower oil

Removing of tocopherols by adsorption chromatography led to oxidation of tocopherol-stripped sunflower oil. The decrease of linoleic and linolenic acids (Table 1) was evident from fatty acid composition. The total tocopherol content was considerably reduced from 149.0 to 8.7 mg/kg (Table 1). Oxidative stability of tocopherol-stripped sunflower oil (IP_Rancimat_ = 2.51 h; IP_Oxidograph_ = 2.0 h) decreased in comparison with that of the original sunflower oil (IP_Rancimat_ = 2.94 h; IP_Oxidograph_ = 3.3 h) due to a decrease in the level of naturally present tocopherols. This method of removing of tocopherols from oil is not suitable for two reasons: relatively high amounts of the residue remains in test medium and some oxidation changes of test medium can be assumed, in contrast with the argument of Waraho et al. [18].

#### 3.1.2 FAME prepared from sunflower oil

Fatty acid composition of FAME was nearly equal to the original sunflower oil but the tocopherol content was found to be zero (Table 1). Oxidative stability of FAME (IP_Rancimat_ = 0.82 h; IP_Oxidograph_ = 0.1 h) decreased significantly in comparison with that of the original sunflower oil (IP_Rancimat_ = 2.94 h; IP_Oxidograph_ = 3.3 h) due to the complete removal of naturally present tocopherols from original sunflower oil. This method of removing of the tocopherols is optimal as it results in zero tocopherol concentration and insignificant oxidation changes.

### 3.2 Methylation of α-, γ-, and δ-tocopherol by diazomethane (CH_2_N_2_)

There is a hypothesis that a hydroxyl group in the tocopherol molecule could be protected by methylation [25] and thus the antioxidant effect of naturally present tocopherols might be eliminated.

It was found by GC-FID (Table 2) that δ-tocopherol reacts with CH_2_N_2_ more readily in comparison with γ- and α-tocopherol. This fact can be explained by the positive inductive effect of substituents in the tocopherol molecule (three methyl groups in the case of α-tocopherol and two methyl groups in the case of γ-tocopherol). Methyl groups (mainly in *ortho* position to the hydroxyl group) decrease the reactivity of the hydroxyl group. Nevertheless, because the degree of tocopherol methylation was found to be negligible, this method (tested for the purpose to eliminate the antioxidant activity of naturally present tocopherols in sunflower oil) failed.

**Table 2:** Conversion of tocopherol (TO) to tocopherol methyl ether (TO–CH_3_) determined in the 1% reaction mixture after chemical reaction of tocopherols with diazomethane

Tocopherol	TO (%)	TO–CH_3_ (%)
α	99.90 ± 0.10	0.10 ± 10^−3^
γ	99.80 ± 0.10	0.20 ± 10^−3^
δ	99.00 ± 0.10	1.00 ± 10^−3^

### 3.3 Comparison of two different methods used for determining the oxidative stability

Oxidograph and Rancimat methods are screening methods with shorter analysis time, the results relate with traditional parameter of lipid oxidation such as peroxide value at 20°C [31]. Introduction of these methods brought about a significant progress in prediction of induction periods during lipid autoxidation and in antioxidant research at the end of 20th century. Especially, the Rancimat method displaced the previously widely used and normalized Schaal oven test.

There are some differences between the Oxidograph and Rancimat methods. During the Oxidograph method the oil sample is measured at 110°C and oxygen atmosphere is formed over the sample (oxygen concentration is high and interface between the sample and the oxygen atmosphere is small), whereas during the Rancimat method the oil sample is measured at 120°C and bubbled by air (oxygen concentration is lower and interface between sample and air is large). The Rancimat temperature of 120°C was chosen according by Mateos et al. [32] in order to obtain similar values of induction periods (to shorten analysis time) as in the case of Oxidograph method.

**Figure 2:**
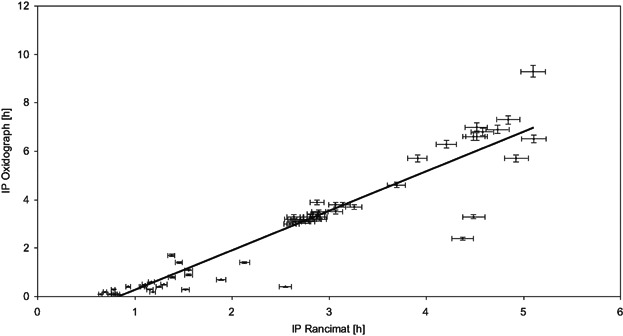
Linear correlation between the induction periods determined by Rancimat method (IP_Rancimat_) and by Oxidograph method (IP_Oxidograph_) − IP_Oxidograph_ (h) = 1.6433 IP_Rancimat_ (h) − 1.4122; *r* = 0.890 (*p*<0.05). Data used were obtained as results of all experiments. Regression analysis was carried out considering all the data (*n* = 68). Error bars express SD (*n* = 3).

Despite of the different principles and conditions of both instrumental methods, a linear correlation was evaluated between induction periods determined by both the Oxidograph and Rancimat method (Fig. 2) in the same lipid matrix. On the basis of this correlation it is suggested that the used antioxidants have very low or no volatility at Rancimat method conditions. The antioxidant volatility significantly decreases the induction period [33].

### 3.4 Antioxidant effect of phenolic acids and their alkyl esters

**Figure 3:**
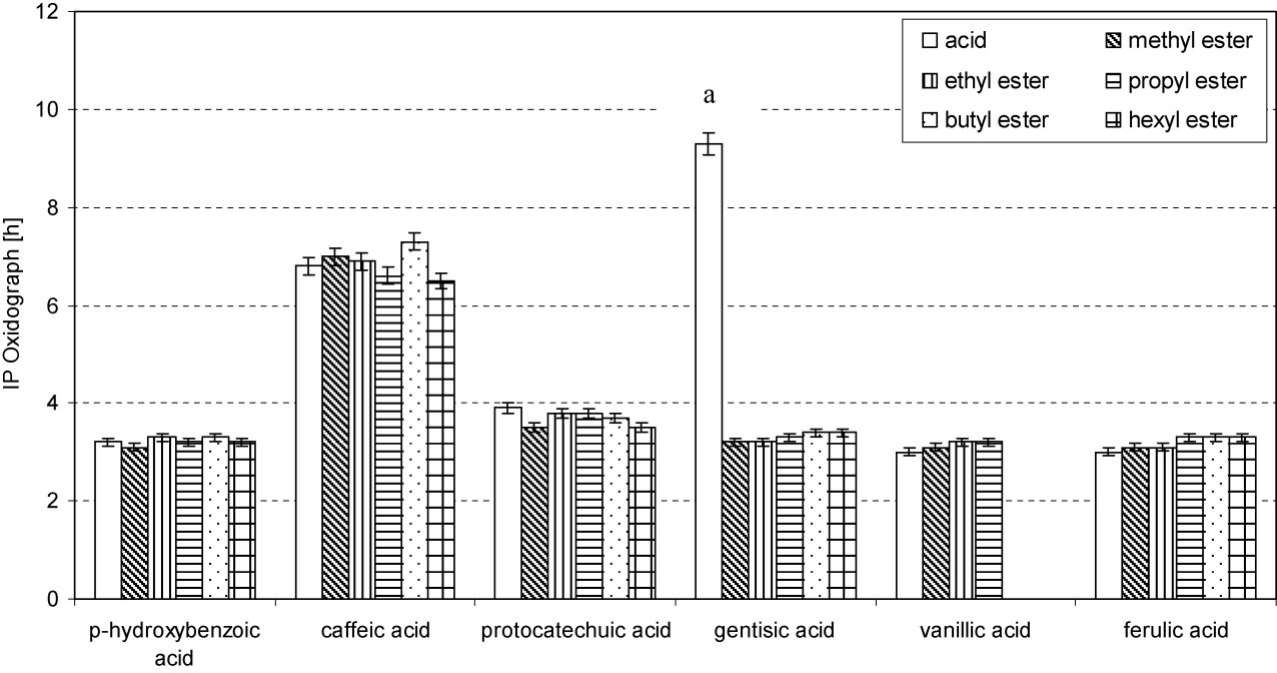
Oxidative stability of phenolic acids and their alkyl esters expressed as induction periods measured in original sunflower oil (total tocopherols 149.0 mg/kg) using the Oxidograph method (IP of original sunflower oil as a blank = 3.3 h). Error bars express SD (*n* = 3). ^a^indicates significant difference (*p*<0.05).

When measured in original sunflower oil containing the common levels of natural tocopherols (Fig. 3), caffeic acid and its alkyl esters exhibited significant antioxidant activity, while the antioxidant activity of protocatechuic acid and its alkyl esters was lower. In the case of both 3,4-dihydroxyphenolic acids (caffeic and protocatechuic acids), no statistically significant differences were found between antioxidative properties of acids and their alkyl esters. The antioxidant effect of gentisic acid alone (2,5-dihydroxyphenolic acid) was the greatest of all studied antioxidants. In contrast, alkyl esters of gentisic acids showed lower antioxidant activity. The explanation for this behavior might be that the strong antioxidant effect of gentisic acid is caused either by synergism with naturally present tocopherols in the original sunflower oil or by the formation of hydrogen bonds between the carboxylic group and hydroxyl group in position 2 in the molecule of gentisic acid.

**Figure 4:**
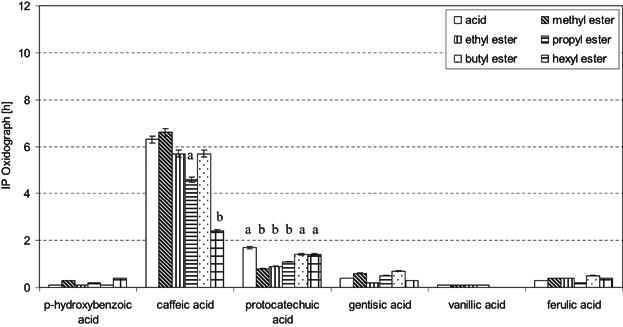
Oxidative stability of phenolic acids and their alkyl esters expressed as induction periods measured in FAME prepared from original sunflower oil (tocopherol-free test medium) using the Oxidograph method (IP of FAME as a blank = 0.1 h). Error bars express SD (*n* = 3). ^a,b^different letters in the same group indicate significant differences (*p*<0.05).

When measured in tocopherol-free FAME prepared from original sunflower oil (Fig. 4), caffeic acid and its alkyl esters had the highest antioxidant activity of all studied antioxidants. Caffeic acid showed a similar antioxidant effect as its methyl, ethyl, and butyl esters, whereas propyl and hexyl esters of the caffeic acid had significantly lower effects. In emulsion systems a cut-off effect was reported. Antioxidant effect of phenolipids with different chain length is changed nonlinearly which is explained by different antioxidant location [12, 13]. In FAME (a nonpolar system) antioxidants with shorter chain length occur on oil–air interface (the critical point is butyl ester of caffeic acid), while antioxidants with longer chain length are dissolved in the oil phase. Generally, the antioxidant activity of alkyl esters of caffeic acid decreased with increasing length of their alkyl chain in conformity with the polar paradox hypothesis [5]. Protocatechuic acid and its alkyl esters exhibited a considerable but much lower antioxidant activity. The antioxidant activity of protocatechuic acid, as opposed to caffeic acid, was observed to be higher than that of its alkyl esters, the antioxidant activity of which increased with increasing length of their alkyl chain. Gentisic acid had a similar antioxidant effect as its alkyl esters. Based on the results obtained, it can be concluded that there is a significant synergistic effect between gentisic acid and naturally present tocopherols in the original sunflower oil, which is evident in Fig. 3, where the extraordinarily high antioxidant activity of gentisic acid can be seen.

**Figure 5:**
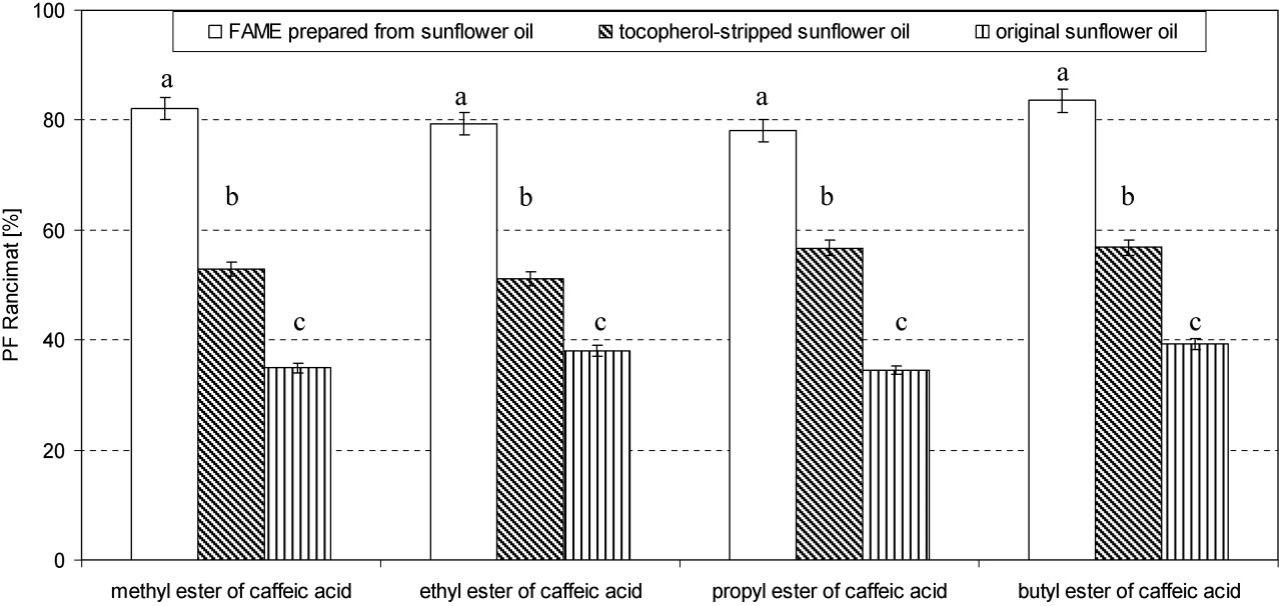
Protection factors of methyl, ethyl, propyl, and butyl esters of caffeic acid measured in FAME prepared from original sunflower oil (tocopherol-free test medium), in tocopherol-stripped sunflower oil (total tocopherols 8.7 mg/kg) and in original sunflower oil (total tocopherols 149.0 mg/kg) using the Rancimat method. Error bars express SD (*n* = 3). ^a,b,c^different letters in the same group indicate significant differences (*p*<0.05).

Methyl, ethyl, propyl, and butyl esters of caffeic acid were chosen as the most effective antioxidants of all antioxidants studied. Their protection factors were determined using the Rancimat method in original sunflower oil (tocopherol content 149.0 mg/kg), in tocopherol-stripped sunflower oil (tocopherol content 8.7 mg/kg) and in FAME prepared from original sunflower oil (tocopherol-free medium). Protection factors of all alkyl esters of caffeic acid decreased with increasing concentration of tocopherols in the respective test medium (Fig. 5), which indicates that naturally present tocopherols in sunflower oil have an antagonistic effect on the antioxidative properties of alkyl esters of caffeic acid.

## 4. Conclusions

Distilled FAME prepared from original sunflower oil may serve as an optimal tocopherol-free test medium for determining the antioxidant activity of various antioxidants. The chemical reaction of tocopherols with diazomethane tested for the purpose of eliminating their antioxidant activity failed due to the negligible degree of methylation of hydroxyl groups in the tocopherol molecules.

3,4-Dihydroxyphenolic acids (caffeic and protocatechuic acids) were evaluated to be more active antioxidants than monohydroxyphenolic acid (*p*-hydroxybenzoic acid), 2,5-dihydroxyphenolic acid (gentisic acid), and 3-methoxy-4-hydroxyphenolic acids (vanillic and ferulic acids).

Naturally present tocopherols in the original refined sunflower oil proved to have a synergistic effect on gentisic acid, whereas gentisic acid measured in tocopherol-free FAME had insignificant effect.

On the other hand, tocopherols appeared to have an antagonistic effect on alkyl esters of caffeic acid, because their protection factors decreased with increasing concentration of tocopherols in the respective test medium. Moreover, their antioxidant activity decreased with increasing length of alkyl chain in conformity with the polar paradox hypothesis.

## Abbreviations

FAME: fatty acid methyl esters of sunflower oil
FID: flame ionization detector
IP: induction period
PF: protection factor
